# Asymptomatic *Plasmodium falciparum* carriage and clinical disease: a 5-year community-based longitudinal study in The Gambia

**DOI:** 10.1186/s12936-023-04519-0

**Published:** 2023-03-07

**Authors:** Abdullahi Ahmad, Nuredin Ibrahim Mohammed, Fatou Joof, Muna Affara, Musa Jawara, Ismaela Abubakar, Joseph Okebe, Serign Ceesay, Majidah Hamid-Adiamoh, John Bradley, Alfred Amambua-Ngwa, Davis Nwakanma, Umberto D’Alessandro

**Affiliations:** 1 Medical Research Council Unit The Gambia at London School of Hygiene and Tropical Medicine, P.O Box 273, Banjul, The Gambia; 2 Global Health Institute, University of Antwerp, Gouverneur Kinsbergencentrum, Campus Drie Eiken, Doornstraat 331, 2610 Wilrijk, Belgium; 3 International Public Health Department, Liverpool School of Tropical Medicine, Liverpool, L3 5QA UK; 4 MRC International Statistics and Epidemiology Group, London School of Hygiene and Tropical Medicine, Keppel Street, London, WC1E 7HT UK

**Keywords:** Asymptomatic, Carriage, *Plasmodium falciparum*, The Gambia

## Abstract

**Background:**

Carriers of persistent asymptomatic *Plasmodium falciparum* infections constitute an infectious reservoir that maintains malaria transmission. Understanding the extent of carriage and characteristics of carriers specific to endemic areas could guide use of interventions to reduce infectious reservoir.

**Methods:**

In eastern Gambia, an all-age cohort from four villages was followed up from 2012 to 2016. Each year, cross-sectional surveys were conducted at the end of the malaria transmission season (January) and just before the start of the next one (June) to determine asymptomatic *P. falciparum* carriage. Passive case detection was conducted during each transmission season (August to January) to determine incidence of clinical malaria. Association between carriage at the end of the season and at start of the next one and the risk factors for this were assessed. Effect of carriage before start of the season on risk of clinical malaria during the season was also examined.

**Results:**

A total of 1403 individuals—1154 from a semi-urban village and 249 from three rural villages were enrolled; median age was 12 years (interquartile range [IQR] 6, 30) and 12 years (IQR 7, 27) respectively. In adjusted analysis, asymptomatic *P. falciparum* carriage at the end of a transmission season and carriage just before start of the next one were strongly associated (adjusted odds ratio [aOR] = 19.99; 95% CI 12.57–31.77, p < 0.001). The odds of persistent carriage (i.e. infected both in January and in June) were higher in rural villages (aOR = 13.0; 95% CI 6.33–26.88, p < 0.001) and in children aged 5–15 years (aOR = 5.03; 95% CI 2.47–10.23, p =  < 0.001). In the rural villages, carriage before start of the season was associated with a lower risk of clinical malaria during the season (incidence risk ratio [IRR] 0.48, 95% CI 0.27–0.81, p = 0.007).

**Conclusions:**

Asymptomatic *P. falciparum* carriage at the end of a transmission season strongly predicted carriage just before start of the next one. Interventions that clear persistent asymptomatic infections when targeted at the subpopulation with high risk of carriage may reduce the infectious reservoir responsible for launching seasonal transmission.

**Supplementary Information:**

The online version contains supplementary material available at 10.1186/s12936-023-04519-0.

## Background

Although malaria burden across sub-Saharan Africa has decreased significantly over the last two decades owing to the scale-up of control interventions [1, 2], malaria transmission has not been interrupted in most endemic areas despite high coverage of control interventions [3]. In endemic settings where clinical immunity to malaria is maintained, a high proportion of infections are clinically silent [4, 5] and, therefore, tend to persist untreated [6]. Such infections remain a constant source of infection to mosquitoes, thus maintaining transmission and hindering control efforts and the progress towards malaria elimination [7, 8].

In the Sahel sub-region, *Plasmodium falciparum* remains the predominant malaria species [9] with transmission occurring mainly during and around the 3 to 4 wet season months and almost ceasing during the long dry season due to diminished mosquito population [10, 11]. A subset of individuals infected at the end of each transmission season however could remain infected without any symptom for several months of the dry season until the start of the following wet season [12], when they probably infect newly emerging mosquitoes thus initiating the yearly seasonal malaria transmission in the community [6, 12]. Across different transmission settings, only few studies have assessed the dynamics of persistent *P. falciparum* carriage [13, 14], thus the extent of carriage and characteristics of carriers are not defined in most endemic areas. As these would likely vary according to the transmission setting [13], identifying risk factors or subgroups associated with persistent *P. falciparum* carriage specific to each setting could guide the targeting of interventions to reduce the human reservoir of infection.

Chronic carriage of multiclonal asymptomatic *P. falciparum* infections has been shown to confer protection against subsequent episodes of clinical malaria [15–19], probably being the result of exposure-dependent enhancement of acquired immunity [20]. Therefore, while clearing persistent asymptomatic infections with effective treatment would reduce the infectious reservoir and possibly transmission, it may increase the risk of clinical malaria in individuals cleared of infection [21]. Nevertheless, other studies have reported an increased risk of clinical malaria in chronic asymptomatic carriers [22–24]. These discordant findings suggest the effect of chronic asymptomatic carriage on protective immunity may vary by transmission setting.

In eastern Gambia, persistent carriage of asymptomatic *P. falciparum* infections, associated risk factors and the effect of carriage on clinical malaria was assessed.

## Methods

### Study setting and participants selection

The malaria burden in The Gambia has decreased significantly over the last 20 years [9, 25], although there is still substantial and heterogeneous residual transmission, particularly in the eastern part of the country [26]. Transmission is almost exclusively by *P. falciparum* species and highly seasonal occurring mainly during the rainy season between July to October and shortly after (November to December) [26]. Several control interventions are routinely implemented by the National Malaria Control Programme, namely insecticide-treated nets (ITN), indoor residual spraying (IRS), intermittent preventive treatment during pregnancy (IPTp) and prompt diagnosis and treatment with artemisinin-based combination therapy (ACT); seasonal malaria chemoprevention (SMC) for children 3–59 months old was introduced in 2014.

In the Upper River Region (URR) in eastern part of the country, four villages were selected by convenience sampling: Gambissara (GMB) with a population of about 13,000 (henceforth referred to as semi-urban village) and three smaller villages surrounding it namely Sare-Bondo (SBD), Fula Morie-Boche (FMB) and Sare-Jawbeh (SJB) with approximate populations of 200, 340 and 150, respectively (henceforth collectively referred to as rural villages). In the semi-urban village, compounds were selected randomly from a list of compounds in the ongoing health demographic surveillance system (HDSS) while in the rural villages all compounds were invited to participate. Within selected compounds, approximately 10 individuals were selected by simple random sampling. Individuals that consented (plus assent if applicable) were enrolled and assigned a unique study identification number and an identification card bearing the participant’s photo and study number. Infants less than 6 months old were excluded from the selection.

### Study conduct

Between 2012 and 2016, two cross-sectional surveys per year in relation to the malaria transmission season were carried out: one just before start (June) and the other at the end (January) of the season (for the year 2016, start of season survey was not conducted due to logistical challenges). Only individuals enrolled into the study were sampled at every survey; demographic and clinical information which included history of fever and symptoms suggestive of clinical malaria in the previous 48 h and at time of survey was collected using a structured questionnaire. Axillary temperature was measured by a digital clinical thermometer. A malaria rapid diagnostic test (RDT) (SD Bioline^®^) was performed for participants with suspected clinical malaria based on clinical assessment and if positive were treated according to the national guidelines.

A blood sample was collected by fingerpick for microscopy and for measuring haemoglobin. Thick blood films were stained with 2.5% buffered Giemsa (PH 7.2) for 10–15 min, dried and read independently by two microscopists. If a slide was positive, parasites were counted against 500 white blood cells (WBCs) and parasite densities estimated assuming 8000 WBC per µl. Slides were considered negative after examining 200 high power fields. If the estimation of the parasite count between the two independent microscopists differed by ≥ 20% or if readings were discrepant for positivity, a third microscopist resolved the discrepancy. Asymptomatic *P. falciparum* carriage was defined as asexual parasitaemia of any density detected by microscopy without symptoms suggestive of clinical malaria within the previous 48 h or at time of assessment during survey. Haemoglobin was measured using a HemoCue^®^ photometer (Ångelholm, Sweden) according to manufacturer’s instruction. A study clinic in the health centre of the study area was established where all study participants sought medical care for any illness during the study period. During each malaria transmission season (August to January), a passive case detection (PCD) system was established where suspected cases of clinical malaria were clinically assessed and systematically screened with malaria RDT (SD Bioline^®^). Positive cases were treated according to the national treatment guidelines. Usage of ITN was assessed by asking all participants attending the study clinic, regardless of their illness, if they had slept under an ITN the previous night.

### Sample size

Sample size was based on desired precision for malaria prevalence at the end of the season (January). Assuming this would range between 5 and 10% in the semi urban village and 15% and 20% in the rural villages based on previous pilot survey (Okebe et al., pers. commun.), 1000 individuals in the semi-urban village allowed for estimation of prevalence with the following precisions: 5% (95% CI 3.7–6.5%), 10% (95% CI 8.2–12.0%) and 200 individuals in the rural villages with the precision of 15% (95% CI 10.4–20.7%), 20% (95% CI 14.7–26.2%).

### Data management and statistical analysis

Data were collected onto paper-based case report forms (CRFs) and then double entered in Studytrax (© Sciencetrax LLC, USA) data base. Consistency checks were carried out, data entry errors and discrepancies verified and corrected prior to statistical analysis which was performed using STATA software version 16.0 (Stata Corp, College Station, Texas, USA). Age was categorized into three groups: < 5 years, 5–15 years and > 15 years; anaemia was defined as Hb < 13.0 g/dl. Severity of anaemia was defined as follows: mild (Hb: 11.0–12.9 g/dl), moderate (Hb: 8.0–10.9 g/dl), and severe (Hb: < 8.0 g/dl) [27]. Descriptive statistics are presented for continuous variables (medians and interquartile range) and proportions for categorical variables. Point estimates are presented with 95% confidence intervals. Malaria prevalence was estimated as the proportion of positive participants by microscopy at each survey and incidence as risk of clinical malaria per 1000 cohort population per transmission season. Evidence of linear trend for prevalence of malaria infection from 2012 to 2016 was assessed. Owing to failed convergence of log-binomial regression models, a mixed effects logistic regression model was used to examine the association between carriage at the end of a season and before start of the next one and to determine independent predictors for persistent carriage (i.e. infected both at the end of a season and at the start of the next one). Mixed effects Poisson regression model was used to determine the association between *P. falciparum* carriage before start of the season and incidence of clinical malaria during the following season and to assess effect measure modification of this association by village setting. The relationship between clinical malaria during the season and risk of carriage at the end of the season was assessed using mixed effects logistic regression. Regression analyses were performed from pooled data for the years 2012 to 2015. All models account for clustering at household level and repeated measurement on subjects allowing for random effects at both levels.

### Ethical considerations

This study was approved by the Gambian Government/MRC Joint Ethics Committee (SCC1256). Written informed consent was obtained from all participants; parents/guardians provided written consent for children less than 18 years old. Written assent in addition to parental consent was obtained from children aged between 12 and 17 years.

## Results

### Description of study population

In June 2012, a total of 1403 individuals were enrolled into the cohort—1154 from the semi-urban village (GMB) and 249 from the three rural villages (SBD, FMB and SJW) combined. Age distribution was similar between the semi-urban and the rural villages. However, ethnic group composition differed, with semi-urban village inhabited mostly by Serahules and the rural villages exclusively by Fulas. Females were more represented in the cohort, particularly in the semi-urban village (Table 1).

**Table 1:** Profile of study villages and characteristics of study cohort at enrolment

Villages	GMB	SBD, FMB, SJB
Setting	Semi-urban	Rural
Enrolled individuals	1154	249
Gender n (%), female	727 (62.9)	132 (53.0)
Age (years), median (IQR)	12 (6–30)	12 (7–27)
Age groups (years), n (%)		
< 5	202 (17.5)	41 (16.5)
5–15	462 (40.0)	109 (43.8)
> 15	490 (42.5)	99 (39.8)
Anaemia n (%)		
Mild (11.0–12.9 g/dl)	656 (56.9)	124 (50.2)
Moderate (8.0–10.9 g/dl)	238 (20.6)	50 (20.2)
Severe (< 8.0 g/dl)	4 (0.3)	4 (1.6)
Ethnic groups n (%)		
Bambara	11 (0.9)	–
Fula	7 (0.6)	249 (100)
Mandinka	17 (1.5)	–
Sarahule	1,119 (97.0)	–

GMB: Gambissara, SBD: Sare Bondo; FMB: Fula Morie Boche; SJB: Sare Jawbeh

### Malaria prevalence before start and at the end of transmission seasons

**Fig.1:**
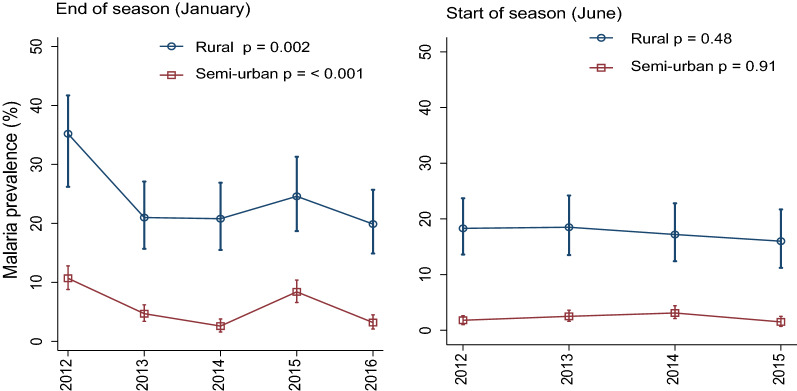
Trend analysis for malaria prevalence at the end and before start of the transmission seasons by village setting. Error bars correspond to 95% confidence interval for estimated prevalence. P values calculated from test for trend of odds of malaria infection. For the year 2016, data was not collected at the start of the season

**Fig. 2:**
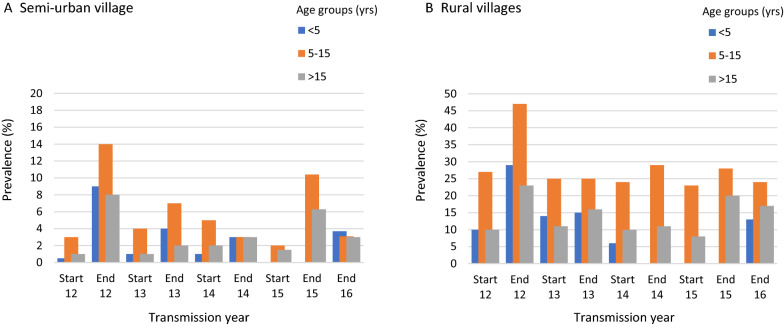
Prevalence of asymptomatic *P. falciparum* malaria by age groups before the start (June) and at end (January) of the transmission seasons. 2012 (abbreviated as 12) to 2016 (abbreviated as 16). Note: (i) For the year 2016, data was not collected at the start of the season. (ii) There are few participants in the < 5 years age group at end of season 2015 survey due to aging of participants over the years of follow up. Additional participants for this age category only were enrolled at end of 2016 survey (Additional file 1: Appendix S7)

Malaria prevalence both just before the start and at the end of the transmission season was consistently higher in the rural villages. Prevalence at the end of the transmission season declined between 2012 and 2016, both in the semi-urban village (from 10.7 to 3.2%) and in the rural villages (from 35.2 to 19.9%) although there was an increase in 2015 within this decline. Overall, prevalence of *P. falciparum* infection at the end of the transmission season showed declining trend over the 5-year study period in both the semi-urban (p < 0.001) and the rural villages (p = 0.002). Remarkably, prevalence before start of the transmission season did not show such declining trend (Fig. 1). Prevalence of infection was consistently higher in the 5–15 years age group, both in the semi-urban and rural villages (Fig. 2). Asexual parasite density tended to be higher in the rural villages (Additional file 1: Appendix S1). Proportion of infected individuals at start of season (June) that harboured gametocytes ranged between 0 and 15.0% in the semi-urban village and between 2.2 and 10.5% in the rural villages (Additional file 1: Appendix S2). Coverage at surveys (proportion of total cohort sampled) ranged between 77.1 and 99.1% and was always slightly higher in the rural villages (Additional file 1: Appendix S3).

In the semi-urban village, compounds with infected individuals just before start of the transmission season varied over the study period (i.e. changed every year); only 1.1% (1/95) of compounds consistently had infected individual(s) at all the four start of season surveys. In the rural villages, 37% (10/27) of compounds consistently had at least one infected individual at every start of season survey particularly in FMB and SJB (Additional file 1: Appendix S4).

The proportion of individuals that reported sleeping under an ITN the previous night during the transmission season estimated from attendees of the PCD clinic over the 5-year study period was slightly higher in the semi-urban (93.8%) than the rural villages (84.8%) (Additional file 1: Appendix S5).

### *Plasmodium falciparum* carriage at end of a season and before start of the following season

**Fig. 3:**
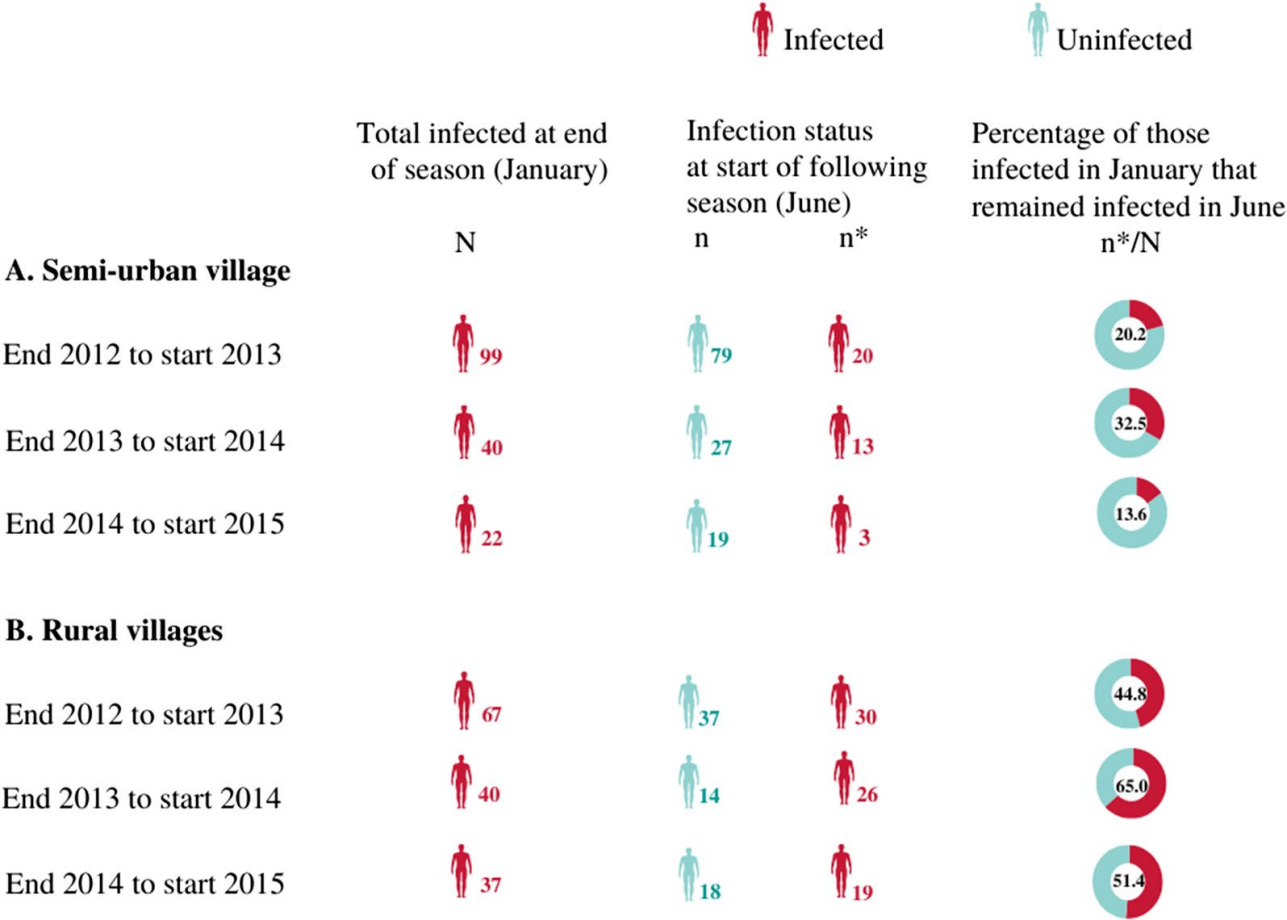
Asymptomatic* P. falciparum* carriage at end of a season (January) and at before start of the following season (June) by village setting. Doughnut chart shows percentage of individuals infected at the end of a season that were again infected at the start of the next season (i.e. persistent carriers)

The proportion of individuals that carried infection at the end of a transmission season and just before start of the next one for the transmission years 2012 to 2015 (i.e. positive at end of 2012 season and at start of 2013, positive at end 2013 and at start of 2014, positive at end 2014 and at start of 2015) was higher in the rural villages each year (Fig. 3). Similarly, majority (95%) of those who carried malaria infection at the end of a season and at start of the next season for more than one year (i.e. multiple years) were from the rural villages. Mixed effect logistic regression analysis from combined data for the years 2012 to 2015 showed a strong association between parasite carriage at the end of the transmission season and at just before start of the next season (OR = 35.04: 95% CI 22.19–55.31; p < 0.001), even after adjusting for age, gender, anaemia, village setting and transmission year (aOR = 19.99; 95% CI 12.57–31.77, p < 0.001). The odds of persistent carriage (i.e. carriage both at the end of the season and at start of the next one) were significantly higher in the rural villages compared to the semi-urban village (aOR = 13.0; 95% CI 6.33–26.68; p < 0.001). Compared to other age groups, children 5–15-year-old had the highest odds of persistent carriage (aOR = 5.03; 95% CI 2.47–10.23; p < 0.001) (Table 2).

**Table 2:** *Plasmodium falciparum* carriage at end of a season and before start of the next season and risk factors for persistent carriage

	Univariate analysis		Multivariate analysis (full model)^a^	
	OR (95% CI)	P value	OR (95% CI)	P value
Association between carriage at the end of a season and before the start of the next season	35.04 (22.19–55.31)	< 0.001	19.99 (12.57–31.77)	< 0.001
Risk factors for persistent carriage				
Village setting				
Semi-urban	1		1	
Rural	9.75 (5.30–17.94)	< 0.001	13.0 (6.33–26.68)	< 0.001
Age groups (years)				
< 5	1		1	
5–15	5.67 (2.82–11.36)	< 0.001	5.03 (2.47–10.23)	< 0.001
> 15	0.77 (0.33–1.81)	0.56	0.74 (0.31–1.78)	0.51
Gender				
Female	1		1	
Male	1.50 (0.95–2.39)	0.08	1.20 (0.73–1.98)	0.45
Anaemia (g/dl)				
No anaemia	1		1	
Mild	1.05 (0.58–1.89)	0.86	1.00 (0.53–1.86)	0.99
Moderate to severe^b^	0.54 (0.26–1.15)	0.11	0.71 (0.31–1.60)	0.41

*OR* odds ratio

^a^Adjusted for age, gender, village setting, anaemia

^b^Merged due to sparse count in the severe anaemia category

### Clinical malaria and association with asymptomatic *P. falciparum* carriage

Incidence of clinical malaria was higher in the rural villages than in the semi-urban village over the study period. Generally, incidence tended to decrease over the study period although there was an increase in 2015 in all study villages (Additional file 1: Appendix S5). Village setting modified the effect of carriage at the start of the season on risk of clinical malaria during the season (p = 0.002 for interaction term carriage-village). While there was no association between carriage at start of the season and risk of clinical malaria during the season in both crude analysis (IRR 1.27, 95% CI 0.83–1.94*,* p = 0.251) and after adjusting for age, gender, years and village setting (IRR 0.81, 95% CI 0.53–1.24, p = 0.348) when rural and semi-urban villages were combined, analysis stratified by village setting showed that parasite carriage before start of the transmission season tended to be associated with a higher risk of clinical malaria during the ensuing season in the semi-urban village (IRR 1.83, 95% CI 0.98–3.42, p = 0.055). Conversely, in the rural villages, parasite carriage just before start of the season was significantly associated with a lower risk of clinical malaria (IRR 0.48, 95% CI 0.27–0.81, p = 0.007) (Table 3). At the compound level, there was no association between asymptomatic carriage in a compound just before start of the season and the risk of subsequent clinical malaria in the same compound, both in the semi-urban (IRR 1.13, 95% CI 0.98–1.30, p = 0.076) and in the rural villages (IRR 0.99, 95% CI 0.88–1.10, p = 0.907).

**Table 3:** Association between carriage of asymptomatic *Plasmodium falciparum* infection before start of the season and risk of clinical malaria during the following season

	Univariate model		Multivariate model^a^	
	IRR (95% CI)	P value	IRR (95% CI)	P value
All village settings combined	1.27 (0.83–1.94)	0.25	0.81 (0.53–1.24)	0.34
Stratified by village setting				
Semi-urban	1.96 (1.05–3.67)	0.035	1.83 (0.98–3.42)	0.055
Rural	0.55 (0.32–0.96)	0.037	0.48 (0.27–0.81)	0.007

*IRR* incidence risk ratio

^a^Adjusted for age, gender, and years

Concerning the risk of asymptomatic carriage at the end of the season after a clinical episode during that season, the association again differed by village setting. In the semi-urban village, the risk of carriage was higher in individuals who had clinical malaria in the preceding transmission months, both in the crude analysis (OR 2.17; 95% CI 1.37–3.42, p < 0.001) and after adjustment for age and gender (aOR 2.09; 95% CI 1.32–3.29, p < 0.001). In the rural villages, this association was reversed as the risk of carriage was significantly lower in individuals who had a clinical malaria in the preceding transmission months both in the crude analysis (OR 0.43; 95% CI 0.24–0.77, p = 0.005) and after adjustment for age, gender and years (aOR 0.39; 95% CI 0.22–0.69, p < 0.001) (Table 4).

**Table 4:** Association between clinical malaria during the transmission season and risk of carriage at the end of the transmission season

	Univariate analysis		Multivariate model^a^	
	OR (95% CI)	P value	OR (95% CI)	P value
All village settings combined	1.32 (0.90–1.94)	0.14	0.90 (0.62–1.31)	0.59
Stratified by village setting				
Semi-urban	2.17 (1.37–3.42)	< 0.001	2.09 (1.32–3.29)	< 0.001
Rural	0.43 (0.24–0.77)	0.005	0.39 (0.22–0.69)	< 0.001

*IRR* incidence risk ratio

^a^Adjusted for age, gender, and years

## Discussion

This study assessed asymptomatic *P. falciparum* carriage at the end of a malaria transmission season and at start of the next one, the risk factors for this and the relationship between carriage and clinical malaria in neighbouring villages of differing transmission intensity. Microscopically patent asymptomatic carriage at the end of the malaria season (January) was strongly associated with carriage just before start of the next season (June); it was estimated that among those infected in January, up to one third in the semi-urban and half in the rural villages could carry infections up to June, when rains usually start, heralding the start of a new malaria transmission season. However, considering infections were identified by microscopy, an important proportion of asymptomatic carriers with sub-patent infections may have been missed [28]. A study conducted in a lower transmission area within the same region that used molecular tests on samples collected monthly during the dry season and, therefore, able to detect sub-patent infections, found that 40% of individuals infected in December remained positive including sub-patent infections until the end of the dry season in May [13]. In the current study, given the known fluctuation of parasitaemia in malaria-infected individuals [28] and the expected decrease in parasite density from partial spontaneous clearance [29], some of those positive in January but negative in June could have been infected at sub-patent level undetectable by microscopy. Therefore, the estimated proportion of individuals that carried infection at the end of a transmission season and at the start of the next one (persistent carriage) reported in this study has probably been underestimated.

The odds of persistent carriage were much higher in rural villages compared to the semi urban village, an observation possibly explained by the higher parasite densities in rural villages which have been shown to persist for longer [29, 30]. Furthermore, given the relatively higher transmission intensity in the rural villages, most infections may have been multiclonal [20], which have been associated with longer persistence [13, 23, 31]. Compared to other age groups, children aged 5–15 years had the highest odds of persistent carriage. This can possibly be due to age-dependent acquisition of immunity that enables older children in endemic areas to tolerate but not eliminate malaria infections [32–34]. Odds of carriage tended to be lower in adults; given that anti-parasite immunity increases with age [34], a faster spontaneous clearance of asymptomatic infections has been observed in adults [29, 35] which could explain this observation. The observed higher odds of persistent carriage in children aged 5–15 years, coupled with the relatively higher exposure of this age group to mosquito bites [36], suggest they probably are major contributors to the initiation of the yearly seasonal malaria transmission in this setting.

The proportion of infected individuals with microscopically patent gametocytes, the parasite stage required for human to mosquito transmission, detected just before start of season was similar to that reported amongst clinical cases during the transmission season [37]. A much higher proportion of gametocyte positive asymptomatic infections has been reported from studies carried out during the dry season that used sensitive molecular diagnostic methods [13, 38]. Remarkably, up to 80% of infections carried to the end of the dry season harboured gametocytes in the study by Collins et al. [13]. Even though gametocyte transmissibility was not assessed in these studies, the findings nonetheless suggest that infections carried through the dry season can potentially infect mosquitoes.

Parasite carriage just before start of the transmission season was associated with the risk of clinical malaria during the following season. However, the direction of such a risk differed between the semi-urban and rural villages. While in the semi-urban village carriage just before start of the season was associated with an increased risk of clinical malaria during the following season, such a risk was decreased in rural villages, a result similar to what has been described in an area of intense transmission of Mali, where individuals with asymptomatic multiclonal infections during the dry season had significantly lower risk of clinical malaria in the following season [17]. Similarly, an earlier study from a high transmission setting in Tanzania observed that baseline multiple infection tended to confer protection against subsequent clinical malaria in older but not in younger children and attributed this to exposure-dependent acquisition of immunity [39]. However, in moderate transmission settings of Senegal [15] and Kenya [31], asymptomatic carriage at the start of the transmission season was associated with an increased risk of clinical malaria as observed in the semi-urban village of the current study. A recent systematic review and pooled analysis of studies concluded that the relationship of asymptomatic infections and risk of subsequent clinical malaria depends on age and transmission intensity; with increasing age, there is a reduced risk in high transmission settings and an increased risk in low to moderate transmission settings [33].

Clinical malaria during the transmission season was associated with the risk of asymptomatic carriage at the end of the season (January); it was significantly lower in rural villages but higher in semi-urban village. Repeated exposure to many heterologous strains in rural villages where transmission is relatively higher would have resulted in an expanded repertoire of relevant memory effector cells, priming the immune system to effectively clear subsequent new infections [20, 40]. In addition, the rural villages were exclusively inhabited by the Fula (Fulani) ethnic group that has been shown to genetically exhibit hyper immune response to *P. falciparum* when exposed [41, 42]. It is also important to point out that infection status was determined by microscopy, therefore, the risk of carriage refers to infection with a relatively high density, detectable by microscopy. Molecular analysis may have provided completely different results as individuals in high and moderate transmission settings may carry very low-density infections. On the other hand, the asymptomatic infections detected after a clinical malaria episode in the semi-urban village may be residual or recrudescent parasitaemia after treatment as has been observed in similar settings [6].

Malaria prevalence at the end of the transmission season declined over the 5-year surveillance period in both the semi-urban and rural villages. This coincided with a relative increased abundance of *Anopheles arabiensis* in the study area (Jawara et al., pers. commun.)*,* a relatively less efficient vector for malaria transmission, which may have contributed to decline in transmission. Findings from recent nationwide entomological survey in The Gambia confirmed predominance of *An. arabiensis* in eastern Gambia over other more efficient vector species [43]. The high coverage of ITNs and IRS which primarily target indoor vector biting in eastern Gambia for an extended period [26] may have exerted selection pressure towards outdoor biting species as observed in Kenya [44] and Senegal [45].

There are a few limitations to consider. Microscopy as diagnostic method has probably underestimated the malaria prevalence and thus the estimated persistence of carriage. In addition, given that microscopy detected infections with a relatively high parasite density while molecular diagnostic methods would have identified low density infections, some of the associations reported above would have been modified had sub-patent infections been included in the analysis. Secondly, in determining persistent carriage, parasitaemia by microscopy was measured at two time points only (i.e. January and June). Even though there is only a remote chance of acquiring a new infection between January and June in this setting, more frequent sampling over the dry season with use of genotyping techniques could have ruled out possibility of new infections with greater certainty. Finally, data on coverage and usage of malaria control interventions was not systematically collected over the 5-year period which limited the interpretation of the study findings in relation to control interventions.

## Conclusions

A significant proportion of individuals infected with malaria at the end of the transmission season carried infection at the start of the following season; carriage was higher amongst children 5–15 years old and in the rural villages. The highly seasonal nature of transmission in this setting suggests such infections persisted throughout the intervening dry season and probably contributed to initiating the next seasonal transmission. Interventions that clear persistent infections when targeted at subpopulations with higher risk of persistent carriage could reduce the malaria infectious reservoir before the emergence of mosquitoes at the start of the rains and may supress the launch of the seasonal malaria transmission in the communities.

## Supplementary Information

**Additional file 1: Appendix S1.** Asexual parasite density (per µ/L) at the start and end of transmission seasons by village setting. **Appendix S2.** Gametocyte carriers (n) among infected individuals (N) at surveys just before start of season (June). **Appendix S3.** Coverage at each cross-sectional survey by village setting n/N (%). **Appendix S4.** Frequency of being an infected compound (at least 1 infected individual in compound) in surveys just before start of transmission (June). **Appendix S5.** Proportion of individuals that slept under insecticide treated net (ITN) the previous night amongst study clinic attendees 2012 to 2016. **Appendix S6.** Incidence of clinical malaria (per 1000 population at risk per season) by village setting. **Appendix S7.** Number of participants by age category sampled at each cross-sectional survey.

## Data Availability

The data used or analyzed in this study are available from the corresponding authors upon reasonable request with approval from the Gambia Government/MRC Joint Ethics Committee.

## Abbreviations

ACT: Artemisinin-based combination therapy
aOR: Adjusted odds ratio
CRFs: Case report forms
Hb: Haemoglobin
HDSS: Health and demographic surveillance system
ICEMR: International Center of Excellence for Malaria Research
IPTp: Intermittent preventive treatment during pregnancy
IRR: Incidence risk ratio
IRS: Indoor residual spraying
ITN: Insecticide-treated nets
MRC: Medical Research Council
OR: Odds ratio
PCD: Passive case detection
RDT: Rapid diagnostic test
SMC: Seasonal malaria chemoprevention
WBCs: White blood cells
