# Supplementary material for: Asymptomatic Plasmodium falciparum carriage and clinical disease: a 5-year community-based longitudinal study in The Gambia
Source: Malar J. 2023 Mar 7;22:82. doi: 10.1186/s12936-023-04519-0 (PMC9993664; doi:10.1186/s12936-023-04519-0)
Supplement: Supplementary file 1 — Additional file 1: Appendix S1. Asexual parasite density (per µ/L) at the start and end of transmission seasons by village setting. Appendix S2. Gametocyte carriers (n) among infected individuals (N) at surveys just before start of season (June). Appendix S3. Coverage at each cross-sectional survey by village setting n/N (%). Appendix S4. Frequency of being an infected compound (at least 1 infected individual in compound) in surveys just before start of transmission (June). Appendix S5. Proportion of individuals that slept under insecticide treated net (ITN) the previous night amongst study clinic attendees 2012 to 2016. Appendix S6. Incidence of clinical malaria (per 1000 population at risk per season) by village setting. Appendix S7. Number of participants by age category sampled at each cross-sectional survey. [file 12936_2023_4519_MOESM1_ESM.docx]

Asymptomatic Plasmodium falciparum carriage and clinical disease: a five-year

community-based longitudinal study in The Gambia

**Ahmad *et al*: Asymptomatic *Plasmodium falciparum* carriage and clinical disease: a five-year community-based longitudinal study in The Gambia.**

**ADDITIONAL APPENDICES**

**Additional appendix 1**:

Asexual parasite density (per µ/L) at the start and end of transmission seasons by village setting.

|  | Asexual parasite density per µ/L  Geometric mean (95% CI) | |
| --- | --- | --- |
|  | Semi-urban village | Rural villages |
| Start of season surveys (June) |  |  |
| 2012 | 43.9 (24.3 – 79.3) | 147.1 (89.4 – 241-9) |
| 2013 | 173.9 (81.4 – 371.4) | 331.1 (198.5 – 552.4) |
| 2014 | 207.4 (112.7 – 381.8) | 329.5 (202.7 – 535.6) |
| 2015 | 61.4 (29.6 –127.5) | 458.4 (289.8 – 724.7) |
| End of season surveys (January) |  |  |
| 2012 | 435.3 (301.6 – 628.23) | 585.6 (372.3 – 921.2) |
| 2013 | 413.2 (229.3 – 744.6) | 598.5 (333.9 –1072.9) |
| 2014 | 312.7 (146.9 – 665.2) | 209.3 (133.3 – 328.3) |
| 2015 | 221.3 (142.4 – 344.1) | 286.4 (176.2 – 465.6) |
| 2016 | 904.7 (357.7 –2288.3) | 533.9 (310.9 – 916.6) |

**Additional appendix 2**:

Gametocyte carriers (n) among infected individuals (N) at surveys just before start of season (June).

|  | Proportion of gametocyte carriage amongst those infected n/N (%). | |
| --- | --- | --- |
| Start of season surveys (June) | Semi-urban village | Rural villages |
| 2012 | 3/20 (15.0) | 1/45 (2.2) |
| 2013 | 2/25 (8.0) | 2/41 (4.9) |
| 2014 | 3/30 (10.0) | 4/38 (10.5) |
| 2015 | 0/13 (0) | 2/33 (6.1) |

**Additional appendix 3**:

Coverage at each cross-sectional survey by village setting n/N (%).

|  |  | 2012  n (%) | 2013  n (%) | 2014  n (%) | 2015  n (%) | 2016  n (%) |
| --- | --- | --- | --- | --- | --- | --- |
| Start of season (June) | Semi-urban village  N=1154 | 1142 (98.9) | 1007 (87.2) | 956 (82.8) | 883 (76.5) | N/A |
|  | Rural villages  N=249 | 247 (99.1) | 222 (89.2) | 221 (88.7) | 206 (82.7) | N/A |
| End of season (January) | Semi-urban village  N=1154 | 1006 (87.2) | 912 (79.0) | 910 (78.8) | 890 (77.1) | 912 (79.0) |
|  | Rural villages  N=249 | 238 (95.5) | 214 (85.9) | 211 (84.7) | 195 (78.3) | 226 (90.7) |

**Additional appendix 4:**

Frequency of being an infected compound (at least 1 infected individual in compound) in surveys just before start of transmission (June).

| Frequency of being an infected compound | None | Once | Twice | Thrice | Quadrice |
| --- | --- | --- | --- | --- | --- |
| Proportion of compounds n/N (%) |  |  |  |  |  |
| Semi-urban Village (N = 95) | 54/95 (56.8) | 23/95 (24.2) | 12/95 (12.6) | 5/95 (5.3) | 1/95 (1.1) |
| Rural Villages (N = 27) | 2/27 (7.4) | 3/27 (11.1) | 9/27 (33.3) | 3/27 (11.1) | 10/27 (37.0) |

**Additional appendix 5:**

Proportion of individuals that slept under insecticide treated net (ITN) the previous night amongst study clinic attendees 2012 to 2016.

|  | Slept under an insecticide treated net (ITN) last night  n/N (%) | |
| --- | --- | --- |
|  | Yes | No |
| Semi-urban village (N=582) | 546/582 (93.8) | 36/582 (6.1) |
| Rural villages (N = 264) | 224/264 (84.8) | 40/264 (15.1) |

**Additional appendix 6:**

Incidence of clinical malaria (per 1000 population at risk per season) by village setting.

| Incidence risk per 1000 cohort population (No of malaria cases / N x 1000) | | | | | |
| --- | --- | --- | --- | --- | --- |
|  | 2012 | 2013 | 2014 | 2015 | 2016 |
| Semi-urban village (N=1154) |  |  |  |  |  |
| No of malaria cases | 66 | 86 | 45 | 76 | 17 |
| Incidence | 57.2 | 74.5 | 39.0 | 65.9 | 14.7 |
| Rural villages (N = 249) |  |  |  |  |  |
| No of malaria cases | 60 | 48 | 27 | 46 | 22 |
| Incidence | 241.0 | 192.8 | 108.4 | 184.7 | 88.4 |

**Additional appendix 7:**

Number of participants by age category sampled at each cross-sectional survey

| **Transmission season surveys** | **Semi-urban village** | | | | **Rural villages** | | | |
| --- | --- | --- | --- | --- | --- | --- | --- | --- |
|  | **Age group (years)** | | | **Total** | **Age group (years)** | | | **Total** |
|  | **< 5** | **5-15** | **>15** |  | **< 5** | **5-15** | **>15** |  |
| Start 2012 | 198 | 458 | 486 | **1142** | 41 | 107 | 99 | **247** |
| End 2012 | 140 | 446 | 420 | **1006** | 34 | 106 | 98 | **238** |
| Start 2013 | 118 | 466 | 423 | **1007** | 28 | 104 | 90 | **222** |
| End 2013 | 73 | 439 | 400 | **912** | 19 | 105 | 90 | **214** |
| Start 2014 | 78 | 461 | 417 | **956** | 18 | 110 | 93 | **221** |
| End 2014 | 37 | 465 | 408 | **910** | 10 | 105 | 96 | **211** |
| Start 2015 | 16 | 476 | 391 | **883** | 9 | 110 | 87 | **206** |
| End 2015 | 0 | 462 | 428 | **890** | 1 | 102 | 92 | **195** |
| Start 2016 | No survey | No Survey | No Survey |  | No Survey | No Survey | No Survey |  |
| End 2016 | 187 | 392 | 333 | **912** | 45 | 97 | 84 | **226** |

Note: i) For Start of season 2016, data was not collected due to logistical challenges. ii) The few participants in the < 5 years age group by end of season 2015 survey was due to aging of participants in this age group over the years of follow up. Additional participants only for the < 5 years age group were enrolled at end of season survey 2016.
